# ﻿Preliminary survey of midget cave spiders (Araneae, Leptonetidae) from forest ecosystems in southern China with the description of three new species

**DOI:** 10.3897/zookeys.1247.154546

**Published:** 2025-07-28

**Authors:** Zhong-Jing Wang, Bin-Lu Liu, Yan-Bin Yao, Ying-Ying Shi, Ke-Ke Liu

**Affiliations:** 1 Key Laboratory of Jiangxi Province for Biological Invasion and Biosecurity, Jinggangshan University, Ji’an 343009, Jiangxi, China Jinggangshan University Ji’an China; 2 Jinshan College of Fujian Agriculture and Forestry University, Fuzhou 350007, Fujian, China Jinshan College of Fujian Agriculture and Forestry University Fuzhou China

**Keywords:** Distribution, leptonetid species, new species, review, taxonomy

## Abstract

This study reports six leptonetid species from three genera collected in karst-associated forest ecosystems of southern China. Three new midget cave spider species are diagnosed, described, and illustrated: *Pararanajiufushanensis* Yao & K. K. Liu, **sp. nov.** (♂, ♀; Fujian Province), *P.songyuani* Yao & K. K. Liu, **sp. nov.** (♂; Guangdong Province), and *Leptonetelazhaoruii* Yao & K. K. Liu, **sp. nov.** (♂, ♀; Fujian Province). Notably, the female of *P.gaofani* Lin & Li, 2022 is described for the first time, while the holotype of *P.mingxuani* Yao & Liu, 2024 is illustrated with detailed images of the male palpal characters. Additionally, *Rhyssoleptonetalishan* Tong, 2024 is newly recorded in Jiangsu Province, expanding its known geographic range. Detailed distribution records are provided for all species, emphasizing southeastern China’s significance as a hotspot for leptonetid diversity and adaptive radiation. These findings refine taxonomic frameworks for karst-associated spiders and enhance understanding of their biogeographic patterns in East Asian karst systems.

## ﻿Introduction

The family Leptonetidae Simon, 1890, comprises small-bodied arachnids highly adapted to cryptic habitats such as caves, rock crevices, and humus layers (Wang et al. 2017, 2020; Liu et al. 2024). Some highly cave-specialized leptonetids (e.g., deep-cave dwellers) exhibit pronounced troglomorphic traits such as eye reduction, depigmentation, and elongated appendages (Wang et al. 2020). However, the species described here retain normal eyes and pigmentation, suggesting an epigean or a less specialized subterranean lifestyle. Globally, 22 genera and over 397 species have been documented (WSC 2025), with diversity hotspots in East Asia, Europe, and North America. Taxonomic delineation in Leptonetidae is particularly challenging due to their small body size, simplified female genitalia, and cryptic morphological diversity. Critical structures (e.g., male median apophyses or female spermathecae stalk) often require high-resolution microscopy to resolve. Recent advances in scanning electron microscopy (SEM), micro-CT imaging, and multilocus molecular phylogenetics (Jochum et al. 2024; Wipfler et al. 2016; Garcia et al. 2017) have significantly enhanced cryptic species identification and evolutionary inference.

China represents a global biodiversity epicenter for Leptonetidae, currently harboring 8 genera and 151 species (Li and Lin 2016; Wang et al. 2020; WSC 2025), predominantly distributed across karst regions in Guangxi, Guizhou, Hunan, Fujian, and Anhui Provinces. Pioneering studies led by researchers from the Institute of Zoology, Chinese Academy of Sciences, have described over 100 new species through integrative taxonomic approaches (Wang et al. 2017, 2020). Recent work demonstrates the efficacy of DNA barcoding (COI gene) coupled with automated barcode gap discovery in resolving morphologically cryptic species (Wang et al. 2020). Biogeographic analyses further reveal divergent evolutionary trajectories between eastern and western Eurasian lineages, implicating Miocene geological events in shaping subterranean dispersal (Wang et al. 2020). Most prior studies on Leptonetidae have focused on cave-dwelling species, while epigean (forest-dwelling) populations in karst regions remain overlooked. Our survey in non-cave habitats reveals a hidden diversity of these understudied lineages.

In recent years, our research team has conducted systematic investigations of epigean ecosystems in Jiangxi, Fujian, Anhui, Guangdong, and Jiangsu Provinces, leading to the discovery of multiple new leptonetid species which led to deeper taxonomic revisions (Liu et al. 2024; this study). In Fujian, systematic sampling and micromorphological analyses of the genus *Longileptoneta* Seo, 2015 revealed seven new species (e.g., *L.guadunensis* Yao & Liu, 2024, *L.huboliao* Yao & Liu, 2024), characterized by significant interspecific differences in male palpal structures (e.g., tibial apophysis morphology, prolateral lobe differentiation) and female genitalia (e.g., spermathecal coiling patterns, epigynal shape) (Liu et al. 2024). Further extending this line of research, this study integrates taxonomic revisions for three leptonetid genera, including descriptions of three new species in *Pararana* Lin & Li, 2022 and *Leptonetela* Kratochvíl, 1978, supplementary morphological characterizations of female *Pararana* specimens, and updated distribution records for *Rhyssoleptoneta* Tong & Li, 2007, thereby refining the systematic framework of East Asian Leptonetidae. These findings further confirm southeastern Chinese pivotal role as a core region for the evolutionary diversification and adaptive radiation of leptonetid spiders, while offering critical data support for studies on the geographic distribution and dispersal history of this spider family.

## ﻿Materials and methods

Specimens were collected under stones on shaded slopes in forest habitats. Specimens were examined using a Jiangnan SZ6100 stereomicroscope with a KUY NICE CCD camera at Key Laboratory of Jiangxi Province for Biological Invasion and Biosecurity, Jinggangshan University. Male and female copulatory organs in this paper were dissected and examined in 80–85% ethanol. The endogynes were dissected with sharp needles and cleaned with pancreatin. All specimens were photographed with an Olympus CX43 compound microscope with a KUY NICE CCD camera. For SEM photographs, specimens were dried under natural conditions, coated with gold using a small ion-sputtering apparatus ETD-2000, or without coating, and examined with a ZEISS EVO LS15 scanning electron microscope.

All measurements were made using a stereomicroscope (AxioVision SE64 rel. 4.8.3) and are given in millimeters. Leg measurements are given as: total length (femur, patella, tibia, metatarsus, and tarsus). All examined specimen materials are deposited in the Animal Specimen Museum, College of Life Science, Jinggangshan University, Ji’an, China (**ASM-JGSU**).

Terminology of the male palp follows Liu et al. (2024) and Li et al. (2024). Abbreviations used in the text or figures are as follows: **ALE** = anterior lateral eye; **At** = atrium; **Con** = conductor; **Em** = embolus; **MA** = medial apophysis; **MO** = median outgrowth; **MS** = median sclerite; **PL** = prolateral lobe; **PLE** = posterior lateral eye; **PME** = posterior median eye; **PS** = prolateral sclerite; **RL** = retrolateral lobe; **Spe** = spermathecae; **SS** = spermathecae stalk; **SPr** = short projection; **SRS** = spine-like retrolateral sclerites; **VS** = ventral sclerite.

## ﻿Taxonomy

### ﻿Family Leptonetidae Simon, 1890

#### ﻿Genus Leptonetela

Taxon classificationAnimaliaAraneaeLeptonetidae

Kratochvíl, 1978

D578DFF3-75E1-5D4E-83E1-E934683A11E1

##### Type species.

*Leptonetelakanellisi* (Deeleman-Reinhold, 1971).

##### Type locality.

Greece, Koutouki Cave near Ljopessi, 11 January 1969.

#### ﻿ Leptonetela zhaoruii

Taxon classificationAnimaliaAraneaeLeptonetidae

Yao & K. K. Liu sp. nov.

EFD7954B-A6AE-5A9F-B2D1-A05A1DF90669

https://zoobank.org/14EF91DF-332B-408E-8514-A7207CDA6E13

Figs 1A−I , 2A−C

##### Type material.

***Holotype***: • ♂, **China**: Fujian Province, Fuzhou City, Jin’an District, Fuzhou National Forest Park, 26°10'14.13"N, 119°16'48.38"E, 26 April 2024, Y. Yao, J. Gong, M. Wu and R. Zhao leg. (Lep-24, ASM-JGSU). ***Paratype***: • 1 ♀, other data as same as the holotype. (Lep-24, ASM-JGSU).

##### Diagnosis.

**Figure 1.**
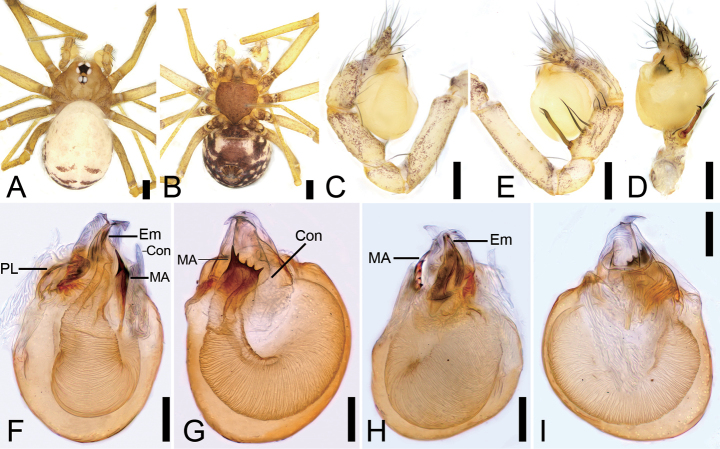
*Leptonetelazhaoruii* sp. nov., male holotype. A. Habitus, dorsal view; B. Same, ventral view; C. Palp, prolateral view; D. Same, ventral view; E. Same, retrolateral view; F. Left bulb, prolateral view; G. Same, ventral view; H. Same, retrolateral view; I. Same, dorsal view. Abbreviations: Con – conductor, Em – embolus, MA – medial apophysis, PL – prolateral lobe. Scale bars: 0.2 mm (A, B), 0.1 mm (C–I).

**Figure 2.**
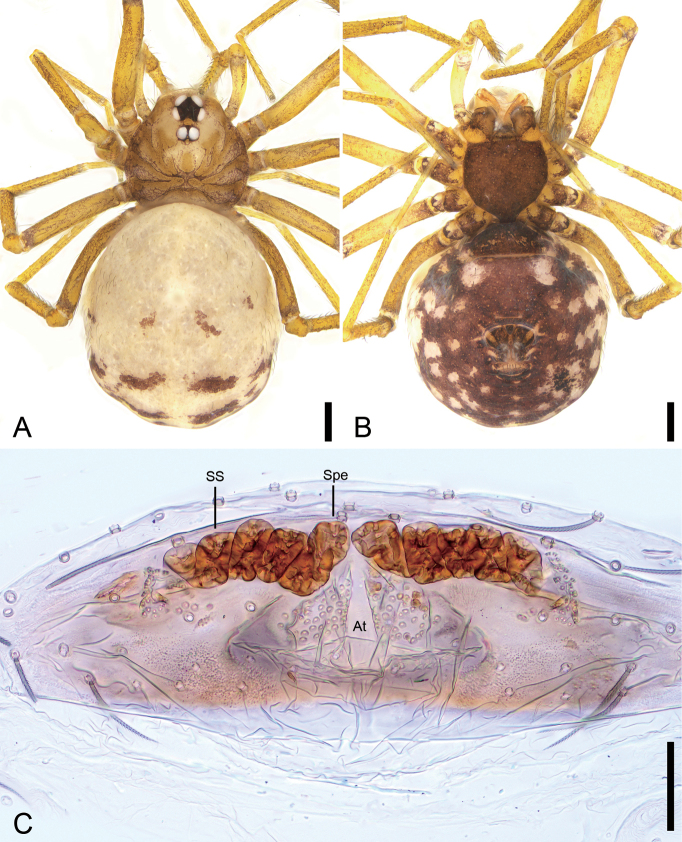
*Leptonetelazhaoruii* sp. nov., female paratype. A. Habitus, dorsal view; B. Same, ventral view; C. Genitalia, dorsal view. Abbreviations: At – atrium, Spe – spermatheca, SS – spermathecae stalk. Scale bars: 0.2 mm (A, B), 0.1 mm (C).

The male of this species is similar to that of *Leptonetelayuanhaoi* Yao & Liu, 2024 (Liu et al. 2024: 293, figs 5B−D, 8E−L) in having the tibia with a row of spines retrolaterally including one thick strong spine proximally and several thin spines, the tongue-shaped prolateral lobe and the median apophysis with five teeth distally but can be separated from it by the median apophysis with broad base (vs narrow) and the prolateral one tooth very strong (vs small) (Fig. 1C−I). The female can be easily distinguished from that of *L.yuanhaoi* (Liu et al. 2024: 293, fig. 6C) by the large subtriangular atrium (vs slightly small and sub-trapezoidal atrium) and ventrally extended spermathecal stalk (vs dorsally) with a small spermathecae (vs slightly enlarged) (Fig. 2C). The female is also similar to that of *L.flabellaris* Wang & Li, 2011 (Wang and Li 2011: 6, fig. 6C) in having the large subtriangular atrium, it can be easily distinguished by the spermathecal stalk with four spirals (vs five) and spermathecae curves anteriorly (vs posteriorly) (Fig. 2C).

##### Description.

**Male (holotype)**. Habitus as in Fig. 1A, B. Total length 1.42, carapace 0.77 long, 0.68 wide. Eye sizes and interdistances (Fig. 1A): ALE 0.07, PME 0.07, PLE 0.08, ALE−PME 0.11, PLE−PLE 0.07, PLE−PME 0.02; AER 0.14, PER 0.18. Clypeus 0.06 high. Chelicerae with eight promarginal (proximal one largest) and four retromarginal teeth. Endites rectangle, with several setae on the upper edge. Labium trapezoid, wider than long. Sternum (Fig. 1B) shield-shaped, longer than wide, anterior margin straight, posterior margin pointed. Legs (Fig. 1A, B): with short setae; measurements: I 3.15 (0.83, 0.25, 0.83, 0.71, 0.53); II 0.9 (0.7, 0.2, missing); III 2.19 (0.59, 0.19, 0.51, 0.54, 0.36); IV 2.99 (0.85, 0.22, 0.72, 0.72, 0.48). Pedicel 0.04. Abdomen (Fig. 1A, B) 2.52 long, 2.62 wide.

***Coloration*** (Fig. 1A, B). Carapace yellow to brown. Chelicerae yellow, with radial ridges along with small brown spots. Endites and labium yellow to brown, with several brown spots. Sternum yellow to dark brown, with numerous small yellow round spots on the surface. Legs yellowish to yellow, coxa and trochanter with some brown spots. Abdomen yellowish to brown, several horizontal brown stripes at the base; venter yellowish to dark brown, with a subtriangular brown stripe medially.

***Palp*** (Fig. 1C−I). Tibia with five long setae retrolaterally, the proximal one very thick, long, strong, spine-like; cymbium with dense setae. Bulb (Fig. 1F−I): prolateral lobe tongue-like, slightly curved, relatively short; median apophysis leaf-shaped, with five teeth, prolateral one very strong with triangular tip, conductor membranous and fan-shaped, covering distal major part of the median apophysis; embolus short, tube-like, slightly curved.

**Female (paratype)**. Habitus as in Fig. 2A, B. As in male, except as noted. Total length 2.19, carapace 0.66 long, 0.64 wide. Eye sizes and interdistances (Fig. 2A): ALE 0.08, PME 0.07, PLE 0.08, ALE−PME 0.10, PLE−PLE 0.09, PLE−PME 0.03; AER 0.15, PER 0.17. Clypeus 0.07 high. Legs (Fig. 2A, B): measurements: I 2.78 (0.76, 0.21, 0.7, 0.59, 0.52); II 2.31 (0.64, 0.23, 0.57, 0.47, 0.4); III 2.13 (0.6, 0.17, 0.49, 0.45, 0.42); IV 2.75 (0.77, 0.22, 0.7, 0.6, 0.46). Pedicel 0.03. Abdomen (Fig. 2A, B) 1.15 long, 0.65 wide.

***Coloration*** (Fig. 2A, B). Darker than male.

***Endogyne*** (Fig. 2C). Internal genitalia with subtriangular atrium, slightly spheroidal spermathecae. Convoluted spermathecal stalk forming three coils.

##### Biology.

Sampled on the woodland floor.

##### Distribution.

Known only from Fujian Province, China (Fig. 13).

##### Etymology.

The species is named after Mr Rui Zhao, who collected the type specimens.

#### ﻿Genus Pararana

Taxon classificationAnimaliaAraneaeLeptonetidae

Lin & Li, 2022

0FF54DE9-9D59-5E2E-B619-012D1B7930D1

##### Type species.

*Pararanagaofani* Lin & Li, 2022.

##### Type locality.

China, Jiangsu Province, Zhenjiang City, Jurong City, Baohua Mountain, 32.1322°N, 119.0915°E, 13 December 2020.

#### ﻿ Pararana gaofani

Taxon classificationAnimaliaAraneaeLeptonetidae

Lin & Li, 2022

BE7B4874-D7EC-5434-80DB-7E6A3421727A

**Figure 3.**
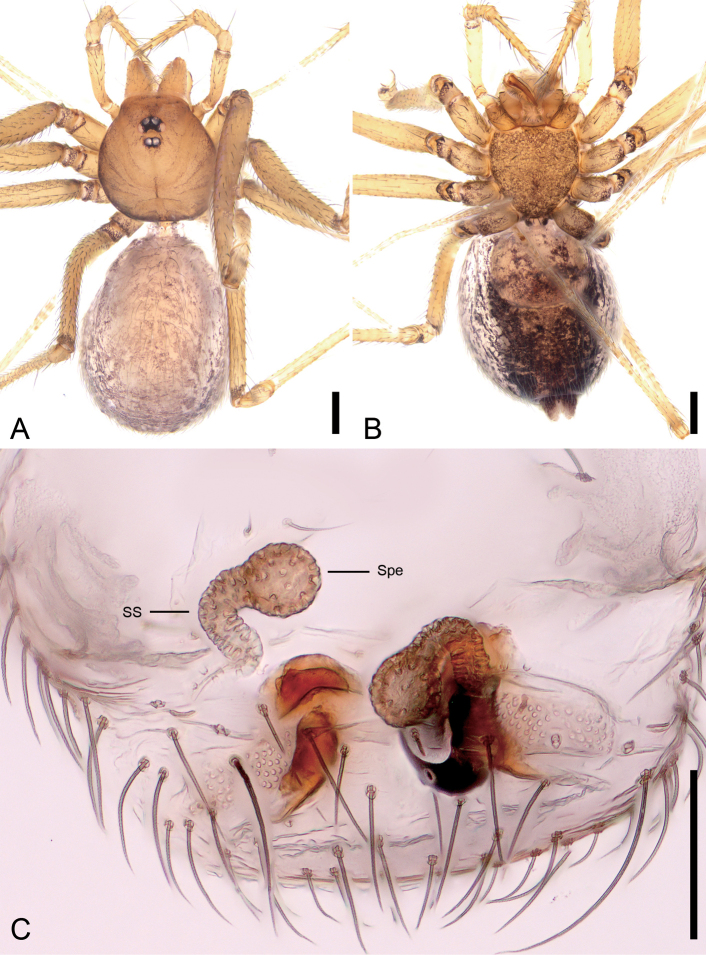
*Pararanagaofani* Lin & Li, 2022, female. A. Habitus, dorsal view; B. Same, ventral view; C. Genitalia, dorsal view. Abbreviations: Spe – spermatheca, SS – spermathecae stalk. Scale bars: 0.2 mm (A, B), 0.1 mm (C).

**Figure 4.**
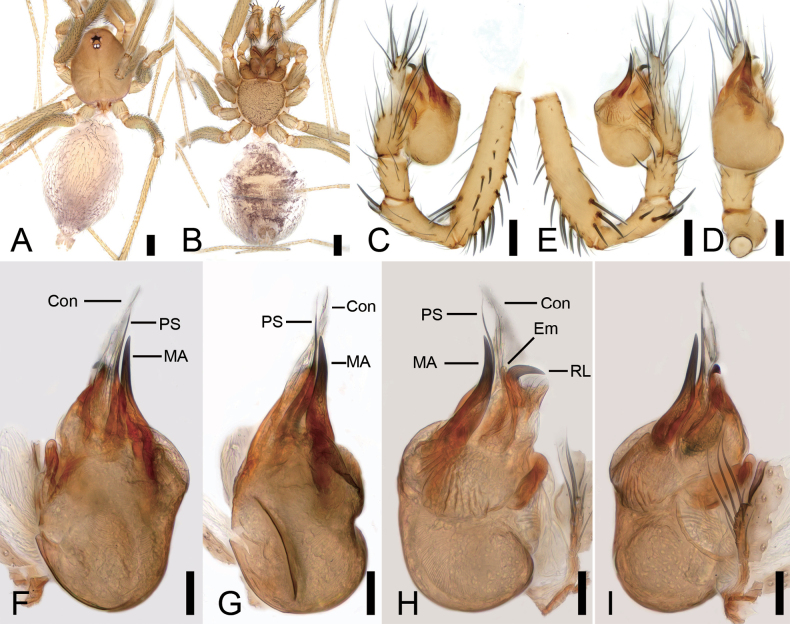
*Pararanagaofani* Lin & Li, 2022, male. A. Habitus, dorsal view; B. Same, ventral view; C. Palp, prolateral view; D. Same, ventral view; E. Same, retrolateral view; F. Left bulb, prolateral view; G. Same, ventral view; H. Same, retrolateral view; I. Same, dorsal view. Abbreviations: Con – conductor, Em – embolus, MA – medial apophysis, PS – prolateral sclerite, RL – retrolateral lobe. Scale bars: 0.2 mm (A, B), 0.1 mm (C–I).

Figs 3A−C , 4A−I , 12A, B

Pararana gaofani Lin & Li, in Lin et al. 2022: 217, figs 17A−C, 18A, B (♂, holotype male from Jiangsu Province).

##### Material examined.

• 1 ♂, 1 ♀, **China**: Anhui Province, Tongling City, Tongguan District, Tianjing Lake Scenic Spot, 30°56'54.15"N, 117°47'53.29"E, 8 January 2025, M. Wu and R. Zhao leg. (Lep-22, ASM-JGSU).

##### Diagnosis.

The female of this species is similar to that of *Pararanamingxuani* Yao & Liu, 2024 (Liu et al. 2024: 317, fig. 27C) in having spheroidal spermathecae, but can be distinguished from it by the slightly vertical spermathecal stalk (vs S-shaped spermathecal stalk including two turns) (Fig. 3C).

##### Description.

**Female.** Habitus as in Fig. 3A, B. Total length 1.82, carapace 0.72 long, 0.61 wide. Eye sizes and interdistances (Fig. 3A): ALE 0.05, PME 0.03, PLE 0.04, ALE−PME 0.08, PLE−PLE 0.08, PLE−PME 0.04; AER 0.1, PER 0.15. Clypeus 0.09 high. Chelicerae with seven promarginal and five retromarginal teeth. Endites oval-shaped, longer than wide, with eight club-shaped setae on the upper inner side and dense long setae on the lower inner side. Labium nearly semicircular, wider than long, with a few setae on the upper side. Sternum (Fig. 3B) shield-shaped, nearly as long as wide, with a few setae. Legs (Fig. 3A, B): with short setae; measurements: I 5.03 (1.35, 0.24, 1.48, 1.17, 0.79); II 3.82 (1.06, 0.25, 1.02, 0.81, 0.68); III 3.05 (0.79, 0.21, 0.8, 0.69, 0.56); IV 4.26 (1.23, 0.21, 1.35, 1.00, 0.47). Pedicel 0.06. Abdomen (Fig. 3A, B) 1.04 long, 0.65 wide.

***Coloration*** (Fig. 3A, B). Carapace yellow to brown, outer edge darker, with several radially arranged dark brown stripes on both sides. Chelicerae yellow. Endites and labium dark yellow. Sternum yellow to dark brown, with many ruleless dark brown spots on the surface. Legs yellow to brown, coxa and trochanter with some brown spots. Abdomen yellowish to brown, with dense brown patches and spots; venter yellow to black, with large areas of brown and black spots.

***Endogyne*** (Fig. 3C). Internal genitalia with spheroidal spermathecae, and slightly vertical spermathecal stalk.

***Note*.** The right side of the female epigyne was damaged during preparation, but the remaining structures (e.g., spermathecae, spermathecae stalk) on the left side remain intact and allow for morphological identification.

**Male.** See Lin et al. (2022); habitus is shown in Fig. 4A, B and the palp is shown in Fig. 4C–I.

##### Distribution.

Known from Jiangsu and Anhui Provinces, China (Fig. 13).

#### ﻿ Pararana jiufushanensis

Taxon classificationAnimaliaAraneaeLeptonetidae

Yao & K. K. Liu sp. nov.

496EBC75-5C9C-5BA0-9582-C4863C830968

https://zoobank.org/565D2186-689F-4044-8BC6-D47235676CE9

**Figure 5.**
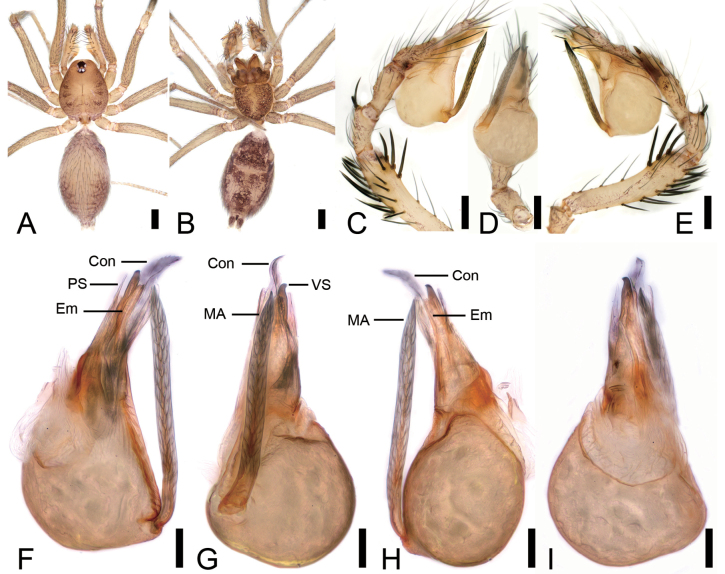
*Pararanajiufushanensis* sp. nov., male holotype. A. Habitus, dorsal view; B. Same, ventral view; C. Palp, prolateral view; D. Same, ventral view; E. Same, retrolateral view; F. Left bulb, prolateral view; G. Same, ventral view; H. Same, retrolateral view; I. Same, dorsal view. Abbreviations: Con – conductor, Em – embolus, MA – medial apophysis, PS – prolateral sclerite, VS – ventral sclerite. Scale bars: 0.5 mm (A, B), 0.1 mm (C–I).

**Figure 6.**
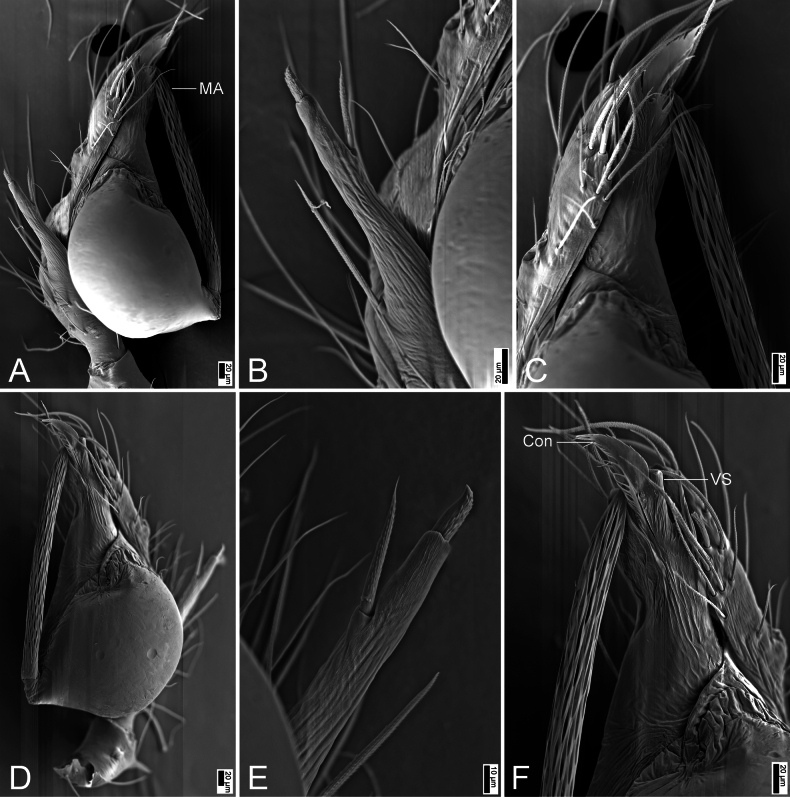
SEM micrographs of *Pararanajiufushanensis* sp. nov., male paratype. A. Right palp, prolateral view; B. Same, detail of the spine on the tibia; C. Same, detail of medial apophysis and conductor; D. Left palp, ventral view; E. Same, detail of the spine on the tibia; F. Same, detail of conductor and ventral sclerite. Abbreviations: Con – conductor, MA – medial apophysis, VS – ventral sclerite. Scale bars: 20 μm (A–D, F), 10 μm (E).

**Figure 7.**
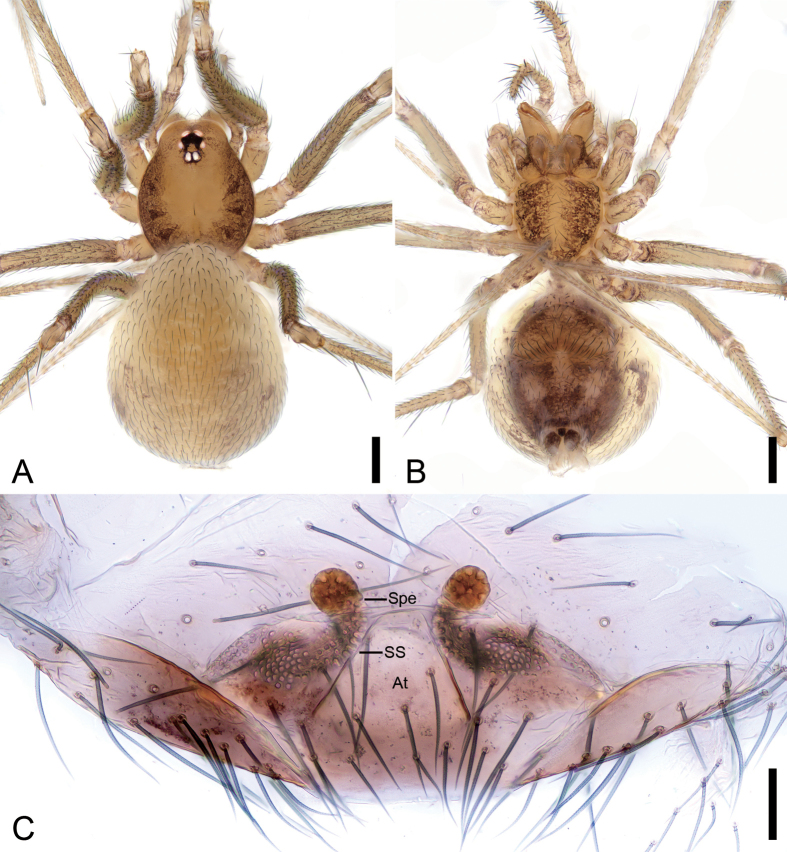
*Pararanajiufushanensis* sp. nov., female paratype. A. Habitus, dorsal view; B. Same, ventral view; C. Genitalia, dorsal view. Abbreviations: At – atrium, Spe – spermatheca, SS – spermathecae stalk. Scale bars: 0.2 mm (A, B), 0.1 mm (C).

Figs 5A−I , 6A−F , 7A−C , 12C, D

##### Type material.

***Holotype***: • ♂, **China**: Fujian Province, Sanming City, Youxi County, Jiufu Mountain Eco-tourism Scenic Spot, 26°6'1.67"N, 118°4'52.25"E, 1 March 2025, Y. Yao, J. Gong, H. Yu, M. Wu and R. Zhao leg. (Lep-25, ASM-JGSU). ***Paratypes***: • 2 ♂, 2 ♀, other data as same as the holotype. (Lep-25, ASM-JGSU).

##### Diagnosis.

The male of this species is similar to that of *Pararanamingxuani* (Liu et al. 2024: 317, figs 25B−D, 26A−E) in having the cymbium with a notch and the swollen patella, but can be easily separated by the patella with two thin and short stick-like spines (vs seven short tooth-like spines), the tibia with a thick and straight spine (vs relatively curved and thin), the thick and highly elongated median apophysis (vs short and thin) and the slightly straight tube embolus (vs curved and rod-like) (Figs 5C−I, 6A−F). The female can be easily distinguished from that of *P.mingxuani* (Liu et al. 2024: 317, fig. 27C) by the sub-trapezoidal atrium (vs bell-shaped) and the C-shaped spermathecal stalk (vs S-shaped) (Fig. 7C).

##### Description.

**Male (holotype)**. Habitus as in Fig. 5A, B. Total length 1.72, carapace 0.66 long, 0.54 wide. Eye sizes and interdistances (Fig. 5A): ALE 0.04, PME 0.05, PLE 0.05, ALE−PME 0.06, PLE−PLE 0.07, PLE−PME 0.02; AER 0.09, PER 0.12. Clypeus 0.11 high. Chelicerae with eight promarginal and five retromarginal teeth. Endites oval-shaped, longer than wide, with 9 club-shaped setae on the upper inner side and dense long setae on the lower inner side. Labium nearly semicircular, wider than long, with a few setae on the upper side. Sternum (Fig. 5B) shield-shaped, nearly as long as wide, with a few setae medially. Legs (Fig. 5A, B): with abundant short setae; measurements: I 1.3 (1.1, 0.2, missing); II 3.13 (0.84, 0.21, 0.83, 0.69, 0.56); III 2.7 (0.77, 0.19, 0.65, 0.61, 0.48); IV 3.74 (1.06, 0.2, 1.04, 0.85, 0.59). Pedicel 0.09. Abdomen (Fig. 5A, B) 0.98 long, 0.55 wide.

***Coloration*** (Fig. 5A, B). Carapace yellow to brown, outer edge darker, with three pairs of radially arranged dark brown patches on both sides. Chelicerae dark yellow. Endites dark yellow to black, with brown spots on the outer margin. Labium dark yellow. Sternum yellow to dark brown, with many ruleless dark brown spots on the surface and with a dark yellow stripe medially. Legs yellowish, with dense small brown spots. Abdomen yellowish to brown, with brown patches along the margin; venter yellow to black, with large areas of scale-like brown and yellow spots.

***Palp*** (Figs 5C−I, 6A−F). Femur with strong spines on each side, ventral view with three long and stout spines especially; patella expanded, with two thin and short stick-like spines; tibia with a very thick spine retrolaterally. Cymbium with a notch subapically. Bulb (Fig. 5F−I): embolus tube-like, middle part slightly curved, located behind the conductor; median apophysis lamellar, extremely long, with a net-shaped surface; conductor membranous, with serrulate margin; Dorsally with two apophysis, one sclerotized and tipped with a hook, the other membranous and pointed.

**Female (paratype)**. Habitus as in Fig. 7A, B. As in male, except as noted. Total length 1.88, carapace 0.66 long, 0.54 wide. Eye sizes and interdistances (Fig. 7A): ALE 0.04, PME 0.05, PLE 0.05, ALE−PME 0.06, PLE−PLE 0.07, PLE−PME 0.03; AER 0.09, PER 0.13. Clypeus 0.09 high. Legs (Fig. 7A, B): measurements: I 3.94 (1.06, 0.21, 1.15, 0.84, 0.68); II 2.87 (0.78, 0.21, 0.75, 0.61, 0.52); III 2.61 (0.72, 0.19, 0.63, 0.58, 0.49); IV 3.61 (0.97, 0.21, 1.05, 0.81, 0.57). Pedicel 0.07. Abdomen (Fig. 7A, B) 1.15 long, 0.65 wide.

***Coloration*** (Fig. 7A, B). Darker than male.

***Endogyne*** (Fig. 7C). Internal genitalia with sub-trapezoidal atrium, slightly spheroidal spermathecae, and C-shaped spermathecal stalk.

##### Biology.

Sampled on the woodland floor.

##### Distribution.

Known only from Fujian Province, China (Fig. 13).

##### Etymology.

The name is taken from the type locality, noun in apposition.

#### ﻿ Pararana songyuani

Taxon classificationAnimaliaAraneaeLeptonetidae

Yao & K. K. Liu sp. nov.

E88FA939-E262-5BDE-B76F-F1B4540FAE66

https://zoobank.org/3BC9DF68-E6AE-4CFD-9205-E7A1A76B3538

Fig. 8A−I

##### Type material.

***Holotype***: • ♂, **China**: Guangdong Province, Guangzhou City, Baiyun District, South Gate of Baiyun Mountain Scenic Area, 23°11'3.37"N, 113°17'59.17"E, 13 February 2024, S. Liu leg. (Lep-21, ASM-JGSU).

##### Diagnosis.

**Figure 8.**
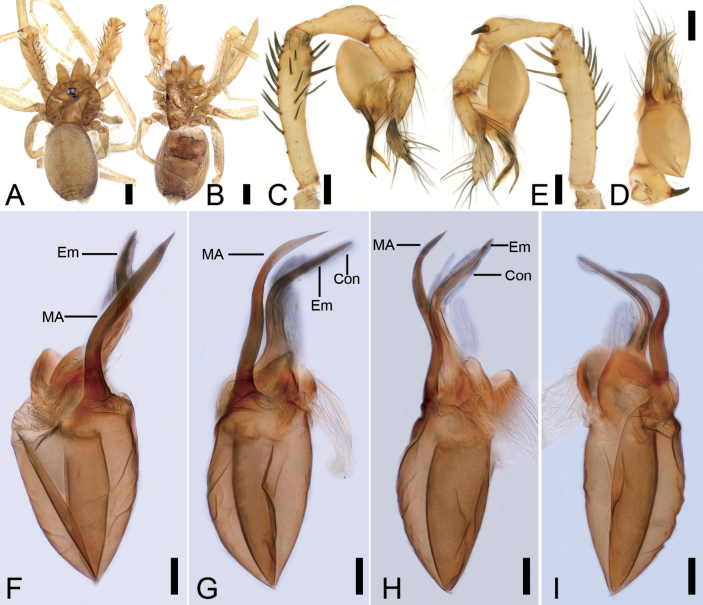
*Pararanasongyuani* sp. nov., male holotype. A. Habitus, dorsal view; B. Same, ventral view; C. Palp, prolateral view; D. Same, ventral view; E. Same, retrolateral view; F. Left bulb, prolateral view; G. Same, ventral view; H. Same, retrolateral view; I. Same, dorsal view. Abbreviations: Con – conductor, Em – embolus, MA – medial apophysis. Scale bars: 0.2 mm (A, B), 0.1 mm (C–I).

The male of the new species is similar to that of *Pararanagaofani* Lin & Li, 2022 (Lin et al. 2022: 217, fig. 17A−C) in having the cymbium with a notch and the swollen patella, but can be easily separated by the patella with only one strong short spine (vs 4 long relatively thin spines), the very long lamellar and curved median apophysis (vs relatively short horn-like median apophysis) and the sclerotized and long embolus (vs membranous and relatively short) (Fig. 8C−I).

##### Description.

**Male (holotype)**. Habitus as in Fig. 8A, B. Total length 1.52, carapace 0.62 long, 0.55 wide. Eye sizes and interdistances (Fig. 8A): ALE 0.07, PME 0.04, PLE 0.07, ALE−PME 0.04, PLE−PLE 0.05, PLE−PME 0.02; AER 0.1, PER 0.14. Clypeus 0.05 high. Chelicerae with nine promarginal and five retromarginal teeth. Endites oval-shaped, with 9 club-shaped setae on the upper inner side. Labium nearly semicircular, wider than long. Sternum (Fig. 8B) shield-shaped, longer than wide. Legs (Fig. 8A, B): with short setae; measurements: I 1.43 (1.22, 0.21, missing); II 3.45 (0.49, 0.19, 0.45, 0.36, 0.2); III 0.98 (0.77, 0.21, missing); IV missing. Pedicel 0.07. Abdomen (Fig. 8A, B) 0. 83 long, 0.57 wide.

***Coloration*** (Fig. 8A, B). Carapace yellow to brown. Chelicerae, endites and labium yellow to dark yellow. Sternum yellow to dark brown, with many ruleless dark brown spots on the surface. Legs yellowish to yellow. Abdomen yellowish to brown, with several brown stripes along the margin; venter yellow to brown.

***Palp*** (Fig. 8C−I). Femur with strong spines on each side, ventral view with three long and stout spines especially; patella expanded, with one strong and short stick-like spines retrolaterally. Cymbium with a notch subapically. Bulb (Fig. 8F−I): embolus tube-like, slender, middle part slightly curved, and then almost parallel to the median apophysis; median apophysis lamellar, extremely long, curves twice into an S-shape, roughly the same length as the embolus; conductor membranous, close adheres to the embolus.

**Female.** Unknown.

##### Biology.

Sampled on the woodland floor.

##### Distribution.

Known only from Guangdong Province, China (Fig. 13).

##### Etymology.

The species is named after Mr Song-Yuan Liu, who collected the type specimens.

#### ﻿ Pararana mingxuani

Taxon classificationAnimaliaAraneaeLeptonetidae

Yao & Liu, 2024

1B12CC87-EE46-5D17-A5AE-3B2E30A855A2

Fig. 9A−D

Pararana mingxuani Yao & Liu, in Liu et al. 2024: 317, figs 25A−D, 26A−E, 27A−C, 28G, H (♂, holotype male from Fujian Province, type deposition in ASM-JGSU).

##### Material examined.

***Holotype***: • ♂, **China**: Fujian Province, Fuzhou City, Yongtai County, Geling Town, Xiyang Village, Tianmen Mountain, 25°49'7.6"N, 119°1'5.07"E, 10.IV.2023, R. Zhao, J. Gong & M. Wu leg. (Lep-9, ASM-JGSU). ***Paratypes***: • 1 ♂, 1 ♀, Fujian Province, Fuzhou City, Minhou County, Nanyu Town, 25°58'24.05"N, 119°13'15.87"E, 5.VI.2023, Y. Yao, W. Zhang, M. Wu & R. Zhao leg. (Lep-9, ASM-JGSU).

##### Description.

**Figure 9.**
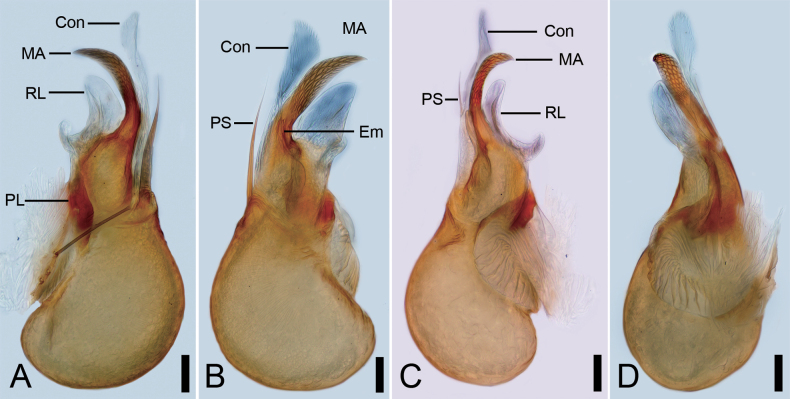
*Pararanamingxuani* Yao & Liu, 2024, male paratype. A. Left bulb, prolateral view; B. Same, ventral view; C. Same, retrolateral view; D. Same, dorsal view. Abbreviations: Con – conductor, Em – embolus, MA – medial apophysis, PL – prolateral lobe, PS – prolateral sclerite, RL – retrolateral lobe. Scale bars: 0.1 mm (A–D).

See Liu et al. (2024), palp as in figs 25B−D, 26A−E. Palpal bulb as in Fig. 9A−D: prolateral lobe banded, long; prolateral sclerite slender, gradually tapers distally, approximately two-thirds the length of the conductor; embolus tube-like, short, slightly curved, with a broad base, behind the conductor; median apophysis lamellar, with a net-shaped surface, almost same length as conductor; conductor membranous, with serrulate margin; retrolateral lobe membranous, crescent.

#### ﻿Genus Rhyssoleptoneta

Taxon classificationAnimaliaAraneaeLeptonetidae

Tong & Li, 2007

BBB9DF00-DDDB-5CB3-BC03-163E37EB0F55

##### Type species.

*Rhyssoleptonetalatitarsa* Tong & Li, 2007.

##### Type locality.

China, Hebei Province, Shijiazhuang City, Zanhuang County, Zhangshiyan Scenic Area, 37.27°N, 114.03°E, 13 September 2005.

#### ﻿ Rhyssoleptoneta lishan

Taxon classificationAnimaliaAraneaeLeptonetidae

Tong, 2024

83F590C9-008E-5CE3-88DB-BEC1D850D4CE

**Figure 10.**
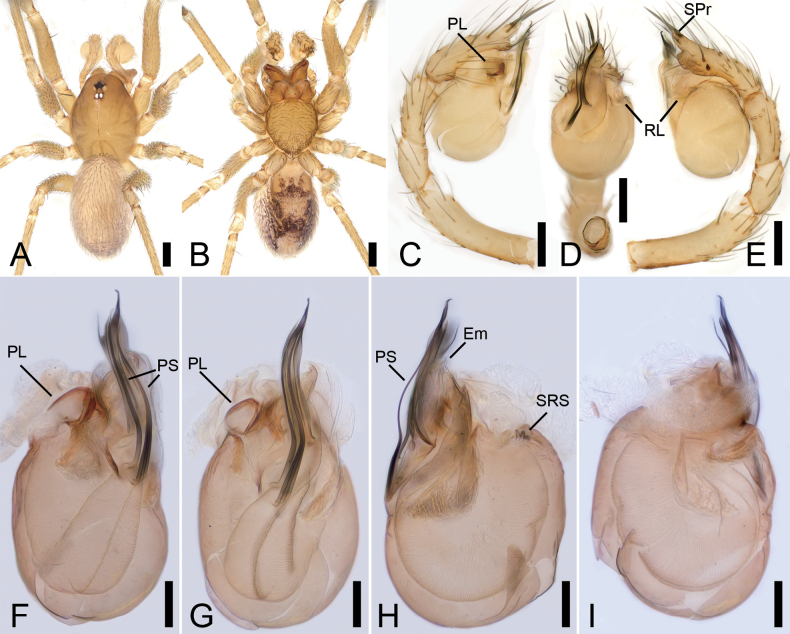
*Rhyssoleptonetalishan* Tong, 2024, male. A. Habitus, dorsal view; B. Same, ventral view; C. Palp, prolateral view; D. Same, ventral view; E. Same, retrolateral view; F. Left bulb, prolateral view; G. Same, ventral view; H. Same, retrolateral view; I. Same, dorsal view. Abbreviations: Em – embolus, PL – prolateral lobe, PS – prolateral sclerite, RL – retrolateral lobe, SPr – short projection, SRS – spine-like retrolateral sclerites. Scale bars: 0.5 mm (A, B), 0.1 mm (C–I).

**Figure 11.**
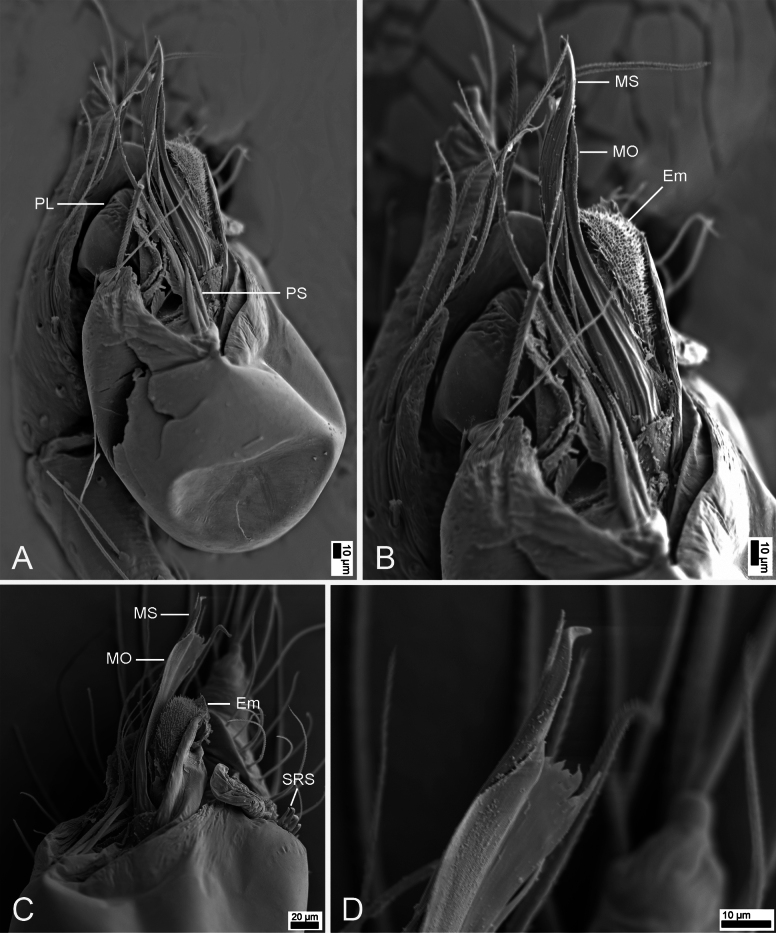
SEM micrographs of *Rhyssoleptonetalishan* Tong, 2024, male paratype. A. Left palp, prolateral view; B. Same, detail of bulb; C. Same, ventral view; D. Same, detail of median outgrowth and median sclerite. Abbreviations: Em – embolus, MO – median outgrowth, MS – median sclerite, PL – prolateral lobe, PS – prolateral sclerite, SRS – spine-like retrolateral sclerites. Scale bars: 10 μm (A, B, D), 20 μm (C).

**Figure 12.**
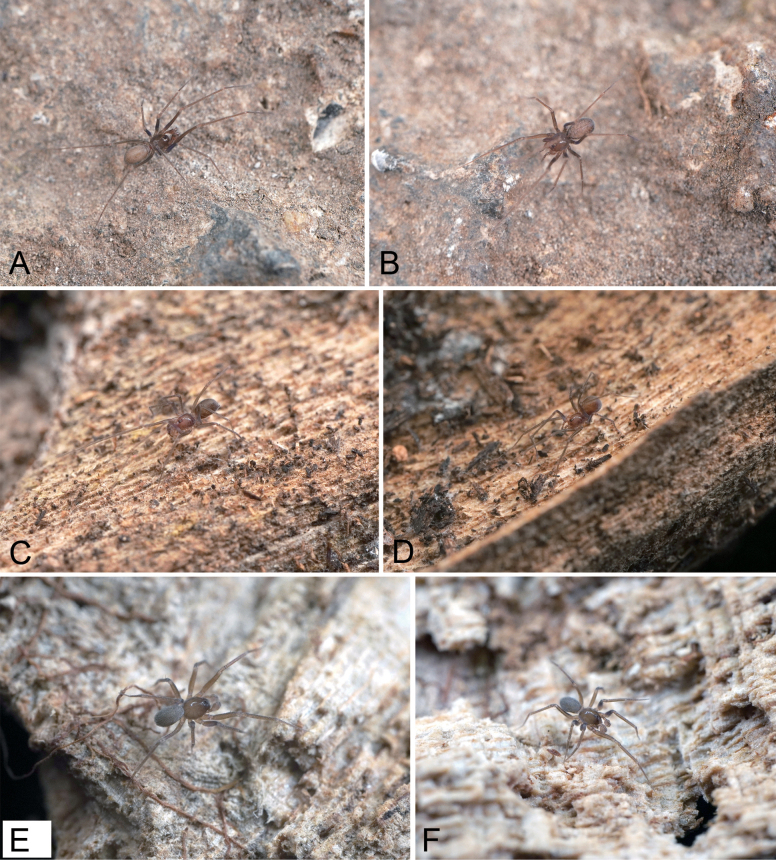
Photographs of living specimens from China. A. *Pararanagaofani* Lin & Li, 2022, male; B. Same, female; C. *P.jiufushanensis* sp. nov., male; D. Same, female; E, F. *Rhyssoleptonetalishan* Tong, 2024, male.

Figs 10A−I , 11A−D , 12E, F

Rhyssoleptoneta lishan Tong, in Li, Chen and Tong 2024: 111, figs 10A−F, 11A−E, 12A−D, 13D (♂, holotype male from Anhui Province).

##### Additional material examined. •

3 ♂, **China**: Jiangsu Province, Wuxi City, Binhu District, Baojie Forest Park, 31°30'44.17"N, 120°14'19.02"E, 22 January 2025, Z. Lyu leg. (Lep-23, ASM-JGSU).

##### Description.

See Li et al. (2024); habitus is shown in Fig. 10A, B and the palp is shown in Figs 10C–I, 11A–D.

##### Biology.

Sampled on the woodland floor.

##### Distribution.

**Figure 13.**
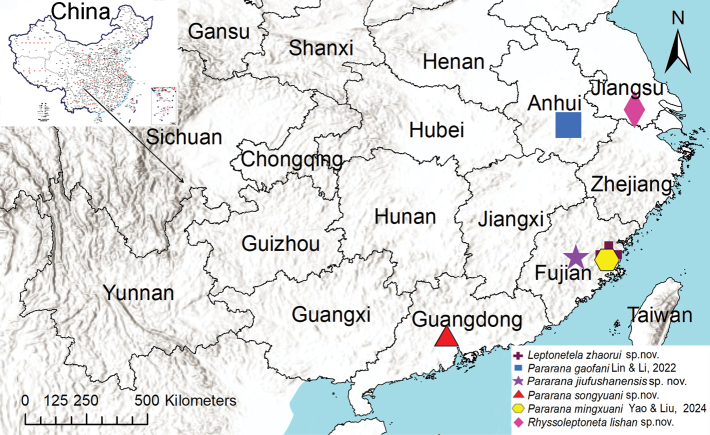
Records of *Leptonetelazhaoruii* sp. nov., *Pararanagaofani* Lin & Li, 2022, *P.jiufushanensis* sp. nov., *P.songyuani* sp. nov., *P.mingxuani* Yao & Liu, 2024, *Rhyssoleptonetalishan* Tong, 2024 from China.

Known from Anhui and Jiangsu Provinces, China (Fig. 13).

## ﻿Discussion

The newly described species *Pararanajiufushanensis* sp. nov. (Fujian) and *P.songyuani* sp. nov. (Guangdong) fill a distribution gap of the genus *Pararana* in South China. Combined with previous records (e.g., *P.gaofani* from Jiangsu and *P.mingxuani* from Fujian) (Lin et al. 2022; Liu et al. 2024), the genus *Pararana* now exhibits a continuous distribution from the middle-lower Yangtze River plain to the Nanling Mountains, suggesting adaptive radiation in humid subtropical karst landscape ecosystems. Notably, the discovery of *Leptonetelazhaoruii* sp. nov. in Fujian further reinforces the role of Guizhou, Guangxi, and other karst regions as global diversity centres for *Leptonetela* (Lin and Li 2010; Wang et al. 2017, 2020), highlighting the potential of South China’s karst landscapes as evolutionary centres of diversification for this group.

The clustered provincial distributions of epigean leptonetids (e.g., *Rhyssoleptonetalishan* in Anhui and Jiangsu; *P.gaofani* in Jiangsu and Anhui) (Lin et al. 2022; Li et al. 2024) could suggest that dispersal may primarily occur through short-range crawling along karst surface fissures. This apparent limited mobility pattern might be associated with their microhabitat specialization in humid rocky substrates, though subterranean water-mediated or long-distance wind-assisted dispersal cannot be ruled out entirely (Elliott et al. 2017). For instance, *Rhyssoleptoneta*, previously known mainly from North China (e.g., *R.aosen* in Beijing and *R.latitarsa* in Hebei; Wang et al. 2020), now extends southward to the lower Yangtze region. While Miocene karst connectivity could have enabled ancestral range expansion, the current distributions of epigean *Rhyssoleptoneta* (e.g., *R.lishan*) (Li et al. 2024) suggest modern dispersal is restricted to localized forest patches, possibly mediated by wind transport or phoresy on vertebrates, with karst fissures acting only as micro-scale corridors.

The first morphological description of the female *P.gaofani* (Lin et al. 2022) reveals critical taxonomic insights. Its C-shaped spermathecal stalk differs distinctly from the S-shaped spermathecal stalk including two turns of *P.mingxuani* (Fujian) (Liu et al. 2024). This discovery not only refines genus-level diagnostic criteria but also underscores the pivotal role of female genital morphology in leptonetid taxonomy. Additionally, the correction made to *P.mingxuani* (i.e., the correct position of embolus and conductor, Fig. 9A−D) clarify boundaries with its congener *P.gaofani* (Lin et al. 2022), providing morphological anchors for future molecular phylogenetic studies.

Although morphological evidence validates the new species, cryptic species complexes persist in Leptonetidae. For example, genetic divergence between Jiangsu and Anhui populations of *R.lishan* (Li et al. 2024), potentially driven by geographic isolation, requires verification via multigene phylogeographic analyses. Furthermore, the concentration of new species in woodland microhabitats (Oh et al. 2022; this study) raises questions about their evolutionary relationships with karst-associated taxa in Guizhou and Guangxi (Wang et al. 2023). Resolving these issues demands systematic sampling across South China and genome-wide phylogenetic reconstructions across multiple taxa to elucidate the origin and radiation pathways of East Asian leptonetid lineages. Such efforts will advance our understanding of karst ecosystems as drivers of subterranean biodiversity.

## Supplementary Material

XML Treatment for Leptonetela

XML Treatment for Leptonetela zhaoruii

XML Treatment for Pararana

XML Treatment for Pararana gaofani

XML Treatment for Pararana jiufushanensis

XML Treatment for Pararana songyuani

XML Treatment for Pararana mingxuani

XML Treatment for Rhyssoleptoneta

XML Treatment for Rhyssoleptoneta lishan

## ﻿Additional information

### Use of AI

No use of AI was reported.

### Author ORCIDs

Zhong-Jing Wang https://orcid.org/0009-0000-6377-6840

Bin-Lu Liu https://orcid.org/0009-0009-3080-8987

Yan-Bin Yao https://orcid.org/0000-0002-2560-9299

Ying-Ying Shi https://orcid.org/0009-0001-1472-5454

Ke-Ke Liu https://orcid.org/0000-0001-7822-3667

### Data availability

All of the data that support the findings of this study are available in the main text.
